# Evaluation of Comorbidities as Risk Factors for Fracture-Related Infection and Periprosthetic Joint Infection in Germany

**DOI:** 10.3390/jcm11175042

**Published:** 2022-08-27

**Authors:** Dominik Szymski, Nike Walter, Volker Alt, Markus Rupp

**Affiliations:** Department of Trauma Surgery, University Medical Centre Regensburg, Franz-Josef-Strauss Allee 11, 93053 Regensburg, Germany

**Keywords:** comorbidities, fracture-related infection, periprosthetic joint infection

## Abstract

Introduction: Fracture-related infections (FRI) and periprosthetic joint infections (PJI) represent a major challenge in orthopedic surgery. Incidence of both entities is annually growing. Comorbidities play an important role as an influencing factor for infection and thus, for prevention and treatment strategies. The aims of this study were (1) to analyze the frequency of comorbidities in FRI and PJI patients and (2) to evaluate comorbidities as causative risk factor for PJI and FRI. Methods: This retrospective cohort study analysed all ICD-10 codes, which were coded as secondary diagnosis in all in hospital-treated FRI and PJI in the year 2019 in Germany provided by the Federal Statistical Office of Germany (Destatis). Prevalence of comorbidities was compared with the prevalence in the general population. Results: In the year 2019, 7158 FRIs and 16,174 PJIs were registered in Germany, with 68,304 comorbidities in FRI (mean: 9.5 per case) and 188,684 in PJI (mean: 11.7 per case). Major localization for FRI were infections in the lower leg (55.4%) and forearm (9.2%), while PJI were located mostly at hip (47.4%) and knee joints (45.5%). Mainly arterial hypertension (FRI: *n* = 3645; 50.9%—PJI: *n* = 11360; 70.2%), diabetes mellitus type II (FRI: *n* = 1483; 20.7%—PJI: *n* = 3999; 24.7%), obesity (FRI: *n* = 749; 10.5%—PJI: *n* = 3434; 21.2%) and chronic kidney failure (FRI: *n* = 877; 12.3%—PJI: *n* = 3341; 20.7%) were documented. Compared with the general population, an increased risk for PJI and FRI was reported in patients with diabetes mellitus (PJI: 2.988; FRI: 2.339), arterial hypertension (PJI: 5.059; FRI: 2.116) and heart failure (PJI: 6.513; FRI: 3.801). Conclusion: Patients with endocrinological and cardiovascular diseases, in particular associated with the metabolic syndrome, demonstrate an increased risk for orthopedic implant related infections. Based on the present results, further infection prevention and treatment strategies should be evaluated.

## 1. Introduction

Infections are one of the most feared complications in orthopedic and trauma surgery. Changes of the demographic structure have led to increased aging of the population and have also resulted in a higher number of patients with acute injuries and chronic diseases of the musculoskeletal system. A lifetime prevalence of 44% for fractures in patients of 55 years or older, and an increase by 14% of fracture incidences (1014/100,000 inhabitants) within the last decade, have demonstrated the relevance of this pathology [1,2].

With an increasing number of fractures, the rate of complications is increasing. One major complication after internal fixation of fractures are infections, which occur in 1–2% of all fractures. The prevalence of fracture related infections (FRI) has increased by 28% within the last eleven years [3]. FRIs represent a risk for the success and positive outcome of fracture treatment. Fracture treatment and elective joint replacements have the risk of complications such as infections, and these represent a huge problem for patients, surgeons and the economy [4,5]. An increasing rate of almost 25% for total knee arthroplasties performed in Germany was reported in the last decade. Likewise, septic revisions due to PJI increased by over 50% within the last decade [6,7]. Similarly, an incidence of PJI between 23.5 and 27.8 per 100,000 inhabitants in Germany was detected and showed constant growth [8]. Both types of infection require long and intense therapy, usually a combination of multiple revision surgeries and long-term antibiotic therapy [9,10]. Even years after infection, a significantly reduced quality of life has been reported despite successful surgical treatment [5,11].

Patient specific risk factors and comorbidities play a relevant role in the development of infections and are a potential approach for prevention. In particular, diseases that modify or weaken the immune system as well as reduce blood flow in the corresponding anatomical region are accountable of increasing infection risk [9,12]. While risk factors for PJIs are well described in the literature, and a clear association has been demonstrated with higher age, increased body-mass-index (BMI) and several comorbidities [13,14,15] for FRI a lack of research is apparent. In PJI, Namba et al. (2012) detected an increased rate of patients with diabetes and a higher rate of ASA (American Society of Anesthesiologists) scores [14]. Additionally, the intake of various drugs modifying the immune system and coagulation were reported with increased infection risk [16].

Such detailed epidemiological information is still missing for FRI, and reveals a lack in the literature. Simultaneously, a comparison between both infection entities in orthopedic surgery has not been obtained, yet. Knowledge of potential causative risk factors, and the evaluation of these, is essential, since patient optimization prior to surgery might help to reduce infections and is therefore of high scientific interest. In particular a comparison between both types of infection with relation to implanted material has not yet been investigated.

Therefore, the aims of this study were to (1) determine frequencies of relevant comorbidities in both FRI and PJI for the first time using the huge database of the German Federal Statistic Office, and (2) compare them between both entities of infection. Furthermore, the comorbidities of FRI and PJI were compared to the occurrence of comorbidities in the general population.

## 2. Materials and Methods

This retrospective study analysed data consisting of annual ICD-10 diagnosis codes from all German medical institutions in the year 2019 provided by the Federal Statistical Office of Germany (Destatis). Patient data of the ICD-10 codes ‘T84.5: Infection and inflammatory reaction caused by a joint endoprosthesis’ and ‘T84.6: Infection and inflammatory reaction due to internal fixation device’ were used to identify hospitalized patients aged 20 years or older diagnosed with PJI or FRI. Health insurance data and hospital billing data are stored in the Federal Statistical Office with details of all coded diagnoses and all procedures performed. The use of data from the Federal Statistical Office allowed full coverage of all cases of FRI and PJI who were in in-patient treatment in Germany in 2019. A detailed analysis of these data with regard to localization and coded secondary diagnosis associated with PJI or FRI were obtained. Localization of FRI was determined through analysis of ICD-10 S-codes, which summarize “injuries, poisoning and certain other consequences of external causes” and allows detection of fractures. For PJIs the ICD-10 codes “Z96.6x—Presence of orthopedic joint implants” was used as a measure for infection localization. Thus, all diseases that were reported, in addition to infection, could be determined from the total data set. For each FRI and PJI, the number of all ICD-10 codes coded in addition to the infection was read out. All diagnoses which were coded in the same case of FRI or PJI were part of the study analysis. In the FRI group, every kind of fracture treatment was considered. No special type of fractures (i.e., pathological fractures) were excluded. For PJI, every reason for primary arthroplasty (post-traumatic degenerative joint disease, primary osteoarthritis, and others.) was included. Classification was obtained according to the ICD-10 codes into organ systems and superordinate diagnosis. Epidemiological studies concerning the general population in Germany were used to compare the occurrence of comorbidities in FRI and PJI patients with the prevalence in the German population. If data from 2019 were not available for the total population, publications as recent as possible (max. three years) were used [17,18,19,20]. To protect the patients and to avoid subsequent assignment of patients, no age or place of residence was given. Categorical data were expressed as frequency counts (percentages). Prevalence of comorbidities was compared between FRI and PJI using the chi-square test. Significance level was set to *p* < 0.05. The relative risk is reported together with the corresponding 95% confidence interval (CI) as the risk compared to the prevalence in the general population in Germany. Prevalence rates were calculated based on Germany’s historical population aged 20 years or older provided by Destatis. Data were analyzed using the statistical software SPSS Version 26.0 (IBM, SPSS Inc. Armonk, NY, USA) and R Statistics Version 4.2.1 (The R Foundation for Statistical Computing, Vienna, Austria).

## 3. Results

**Figure 1:**
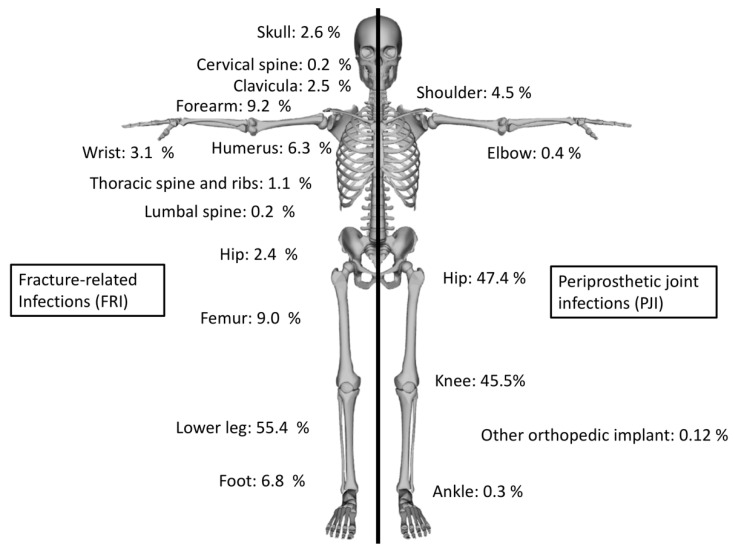
Proportional distribution of the localization of fracture-related infections and periprosthetic joint infections.

In total, 7158 FRIs and 16,174 PJIs were registered in 2019 for hospitalized treatment in Germany. In FRI, 68,304 coded comorbidities (mean: 9.5 per FRI) and in PJI 188,684 (mean: 11.7 per PJI) secondary diagnosis were found. Major localizations for FRI were infections in the lower leg (55.4%), forearm (9.2%) and femur (9.0%). PJIs were most often registered at the hip (47.4%) and knee (45.5%) (Figure 1). Secondary diagnoses among the population of PJI were especially classified as diseases of the cardiovascular (*n* = 26,347; 14.0% of all secondary diagnoses) and endocrine organ system (*n* = 23,364; 12.4%). In patients suffering from FRI, comorbidities were also mainly associated to the cardiovascular system (*n* = 8198; 12.0%) and endocrine diseases (*n* = 6748; 9.9%). Analyzing and comparing in detail coded comorbidities detected in in FRI and PJI patients were mainly arterial hypertension (FRI: *n* = 3645; 50.9% in all cases—PJI: *n* = 11,360; 70.2%; *p* < 0.00001 between FRI and PJI), diabetes mellitus type II (FRI: *n* = 1483; 20.7%—PJI: *n* = 3999; 24.7%; *p* < 0.00001), obesity (FRI: *n* = 749; 10.5%—PJI: *n* = 3434; 21.2%; *p* < 0.00001) and chronic kidney failure (FRI: *n* = 877; 12.3%—PJI: *n* = 3341; 20.7%; *p* < 0.00001) (Table 1). The general population and prevalence of specific comorbidities were used as a reference. In the year 2019, the population of persons older than 20 years was 67,864,036. It was estimated that 6,718,539 persons in Germany were suffering from diabetes mellitus type 2 (9.9%), 21,580,763 from arterial hypertension (31.8%), 12,283,390 had a BMI > 30 and suffering from obesity (18.1%), while 2,307,377 cases of heart failure (3.4%) and 3,936,114 patients with chronic obstructive pulmonary disease (5.8%) were registered. Compared with the general population, an increased risk for PJI was found in patients with diabetes mellitus type 2 (RR: 2.988), arterial hypertension (RR: 5.059) and heart failure (RR: 6.513). In infections following operative treatment of fractures an increased relative risk was found for patients suffering diabetes mellitus (RR: 2.339), arterial hypertension (RR: 2.116) and heart failure (RR: 3.801) (Table 2).

## 4. Discussion

The main finding of this population-based retrospective study were the description of comorbidities in patients with FRIs and PJIs and the evaluation of comorbidities compared to the occurrence in the general population in Germany. Our aim was to detect critical comorbidities in infections after fracture treatment or total joint replacement. In patients with PJI, a higher rate of comorbidities (11.7) per infection was found compared to FRI, with 9.5 comorbidities per case. For heart failure, diabetes mellitus type 2 and arterial hypertension an increased relative risk compared with the general population was found in cases with FRI, while in PJI an increased relative risk (RR) for obesity and chronic obstructive pulmonary disease an increased RR was detected.

Mainly comorbidities of the cardiovascular system were found to be risk factors for PJI and FRI while analyzing all cases with PJI or FRI in Germany provided by the federal statistical office in the year 2019. Compared to the general population, in particular for persons with heart failure or arterial hypertension, an increased relative risk of suffering an FRI or PJI was detected. The RR was almost doubled for both diseases in patients with PJI compared to FRI. The literature already described risk factors for FRIs of different fracture entities in different meta-analysis and literature reviews, but most of them focused on fracture classification and operative treatment. Shao et al. (2017) demonstrated for tibia plateau fractures a higher risk for infections using external fixation, classified as open fracture or a prolonged operation time [21]. Severe soft-tissue defects with delayed wound closure, drug abuse and complex types of factures also showed higher rates for a surgical site infection [22,23,24]. Comorbidities were rarely discussed. A meta-analysis of Peng et al. (2019) concerning surgical site infection in spinal surgery found arterial hypertension to be a risk factor with an odds ratio of 1.52, which lies below the rate our data demonstrated, i.e., 2.1 for FRI and 5.1 for PJI [25]. Deng et al. (2019) also found a higher infection risk for patients with coronary artery disease (*p* = 0.003) after spinal surgery and also found cardiovascular diseases as risk factor for the development of postoperative infections [26]. In addition to the risk factors already described in FRI, such as prolonged duration of surgery, in cases with PJI previous operations, smoking and BMI, peripheral blood circulation situation was also found to be particularly important [9,27,28,29,30]. In our investigation, in particular arterial hypertension and heart failure were associated with PJIs, and showed a relative risk between 5 to 6.5 compared to the general population. Comparing cardiac comorbidities between FRIs and PJIs, a clear trend of increased risk and rates in patients with periprosthetic joint infections was determined.

In addition to cardiovascular diseases, endocrinological causative risk factors play an important role in implant-related bone and joint infections. Metabolic syndrome, with its cluster of syndromes and resulting diseases such as diabetes mellitus, is associated with an increased risk of infection. The function of the immune system in patients with hyperglycemia is impaired and activation and the amount of leukocytes populations is altered. In particular neutrophil cells as well as monocytes populations are limited. Concurrent reduced phagocytosis and bacterial killing mechanisms are described in several publications [31,32,33]. In vitro studies showed increased biofilm presence in models with higher levels of glucose [34] and provided, as well as an impaired immune system and microvascular changes, a reason for the correlation between diabetes and surgical infections [30]. Even in patients without a diagnosed diabetes mellitus but a temporary hyperglycemia increased risk for PJI was detected [35]. For diabetes mellitus an increased relative risk was reported in FRI (2.33) and PJI (2.98) cases. Previous studies have demonstrated a strong correlation between diabetes mellitus and infections for PJIs and FRIs [12,30]. Breznicky et al. (2020) described a relative risk for PJI between 2.2 and 3.5 depending on the therapy of diabetes [27], while Ahmand et al. (2022) reported in their systematic review a 1.8 times increased risk for patients with diabetes mellitus compared to non-diabetic patients after total knee arthroplasty [36]. For FRI, as well, an increased risk with a higher rate of diabetes (*p* = 0.050) in cases with postoperative infection was detected [26]. Additionally, the intake of various drugs such as inhaled corticosteroids (hazards ratio (HR): 2.6), amlodipine (HR: 3.1) and vitamin K antagonists (HR: 5.3) were associated with an increased infection risk after hip arthroplasty [16].

Often, obesity and an increased BMI are associated with diabetes mellitus. In these patients, the duration of an operation is mostly prolonged, and soft tissue is characterized by reduced blood flow, while the patient themselves often suffer from further multiple comorbidities [9,30]. Consequently, an increased risk of infection following surgical procedures in orthopedic and trauma surgery with rates of up to 24% have been described in this difficult to treat patient group [9,12,27,28,30]. However, a BMI < 19 kg/m^2^ has also been described in the literature as a risk factor and should always be considered in preoperative planning [29].

Despite continuous improvements in the pre-, peri- and postoperative treatment of fractures and joint replacements, we are experiencing a steadily increasing rate of infections after surgery [3,7]. The demographic change, the annually growing proportion of patients with metabolic syndrome, further comorbidities and sequelae are potential explanations for this phenomenon. In Germany, more than 50% of the inhabitants are categorized as overweight (BMI > 25 kg/m^2^) at the moment, with an increasing prevalence. In particular with increasing age, an increased prevalence for adiposities has been observed [19]. This part of the population primary constitutes candidates for joint replacement, and should be focused on for infection prevention. However, improved therapy algorithms have been published for the treatment of both FRI [37,38] and PJI [9,30]. A clear classification and sufficient derivation of therapy algorithms can lead to great advantages in the treatment of infections and simplify the treatment process [39].

Our study has several limitations. A major disadvantage is the use of a data register-based ICD-10 query. Only coded diseases, which were used in the cases with FRI and PJI as main diagnosis, could be analyzed. This could use a possible underestimation of comorbidities due to lack of coding or incorrect coding. Due to the query of ICD-10 codes, further causative risk factors, such as age, medication or peri- and postoperative aspects could not be reported. All causes for fractures and arthroplasties were included in the data analysis, which also resulted in further limitation. Traumatic fractures and pathologic fractures were art of the investigation. The same pattern was included in the PJI subpopulation where elective arthroplasties were combined with joint replacements for fracture treatment. However, the aim of the study was to report all comorbidities, and therefore all cases of FRI and PJI were analysed for the year 2019. Another limitation of the comparison with the total population is insufficient matching of the patients’ age structure. Due to the data structure, we could not compare the prevalence of risk factors in both infection groups with the prevalence of an age-matched population. In addition, no statement on the microbiological results could be made. The aim of our comparison was to demonstrate the relative risk of comorbidities in FRI and PJI compared to the general population. For this purpose, the analysis of ICD-10 codes offers the best option to represent all cases of FRI and PJI and their comorbidities in the German population. Due to data assessment by analysis of ICD-10 codes, an underestimation in the quantity of all comorbidities was expectable. For a more detailed analysis of comorbidities and patient related risk factors a nation-wide registry of infections following joint replacements and operative fracture treatment would be necessary.

## 5. Conclusions

In a comparison of PJI and FRI, a higher total number of comorbidities has been documented in PJI cases. Patients with endocrinological and cardiovascular diseases in particular associated with the metabolic syndrome such as arterial hypertension, heart failure and diabetes mellitus demonstrate an increased risk for PJI and FRI in the course of their surgical treatment. The respective comorbidities demonstrated an increased relative risk compared to the normal population, with a higher proportion of PJI. Based on the present results, further infection prevention, optimizing general health and treatment strategies, should be evaluated.

## Figures and Tables

**Table 1:** Absolute number and proportion of comorbidities of fracture-related infections and periprosthetic joint infections with statistical difference between FRI and PJI.

	FRI (ICD-10: T84.6)	PJI (ICD-10:T.84.5)	Significance Level
Total number	*n* = 7158	*n* = 16,174	
	*n* (%)	*n* (%)	*p*-value
Endocrine system			
Diabetes mellitus type I	53 (0.7)	47 (0.3)	*p* < 0.00001
Diabetes mellitus type II	1483 (20.7)	3999 (24.7)	*p* < 0.00001
Obesity	749 (10.5)	3434 (21.2)	*p* < 0.00001
Hypothyroidism	809 (11.3)	2596 (16.1)	*p* < 0.00001
Cardiac system			
Arterial hypertension	3645 (50.9)	11360 (70.2)	*p* < 0.00001
Heart failure	856 (12.0)	3019 (18.7)	*p* < 0.00001
Atrial fibrillation	867 (12.1)	3466 (21.4)	*p* < 0.00001
Pulmonal system			
COPD	391 (5.5)	1118 (6.9)	*p* = 0.000033
Bronchial asthma	179 (2.5)	503 (3.1)	*p* = 0.010846
Urogenital system			
Chronic kidney failure	877 (12.3)	3341 (20.7)	*p* < 0.00001

FRI: Fracture-Related Infection; PJI: Periprosthetic Joint Infection; COPD: Chronic Obstructive Pulmonary Disease.

**Table 2:** Relative Risk and associated confidence interval (CI) of specific comorbidities compared to the general population (older than 20 years in the year 2019).

	FRI (ICD-10: T84.6)	PJI (ICD-10: T.84.5)
Total number	*n* = 7158	*n* = 16,174
Diabetes mellitus type II	2.339 (CI: 2.335–2.343)	2.988 (CI: 2.985–2.992)
Obesity (BMI > 30)	0.521 (CI: 0.52–0.522)	1.22 (CI: 1.218–1.221)
Arterial hypertension	2.116 (CI: 2.163–2.17)	5.059 (CI: 5.053–5.064)
Heart failure	3.801 (CI: 3.792–3.809)	6.513 (CI: 6.505–6.521)
COPD	0.925 (CI: 0.922–0.928)	1.206 (CI: 1.203–1.208)

FRI: Fracture-Related Infection; PJI: Periprosthetic Joint Infection; COPD: Chronic Obstructive Pulmonary Disease.

## Data Availability

Data provided by the German Federal Statistical Office (DESTATIS).
